# Efficiency of dual-energy computed tomography enterography in the diagnosis of Crohn’s disease

**DOI:** 10.1186/s12880-021-00716-y

**Published:** 2021-12-03

**Authors:** Jinghao Chen, Jie Zhou, Jushun Yang, Ruochen Cong, Jinjie Sun, Jing Xiao, Jianhua Shi, Bosheng He

**Affiliations:** 1 Department of Radiology, Affiliated Hospital 2 of Nantong University, Nantong City, 226001 Jiangsu Province China; 2 Department of Radiology, Changzhou Hospital of Traditional Chinese Medicine, Changzhou City, 213000 Jiangsu Province China; 3 Gastrointestinal Surgery, Affiliated Hospital 2 of Nantong University, Nantong City, 226001 Jiangsu Province China; 4 Department of Epidemiology and Medical Statistics, School of Public Health, Nantong University, Nantong City, 226019 Jiangsu Province China; 5 Department of Biochemistry, Nantong University Medical School, Nantong City, 226019 Jiangsu Province China; 6 Clinical Medicine Research Center, Affiliated Hospital 2 of Nantong University, Nantong City, 226001 Jiangsu Province China

**Keywords:** Crohn’s disease, Virtual monoenergetic imaging, Iodine map, Dual energy CT, CT enterography

## Abstract

**Background:**

This retrospective study aimed to investigate the usefulness of the optimized kiloelectron volt (keV) for virtual monoenergetic imaging (VMI) combined with iodine map in dual-energy computed tomography enterography (DECTE) in the diagnosis of Crohn’s disease (CD).

**Methods:**

Seventy-two patients (mean age: 41.89 ± 17.28 years) with negative computed tomography enterography (CTE) were enrolled for investigating the optimized VMI keV in DECTE by comparing subjective and objective parameters of VMIs that were reconstructed from 40 to 90 keV. Moreover, 68 patients (38.27 ± 15.10 years; 35 normal and 33 CD) were included for evaluating the diagnostic efficacy of DECTE iodine map at the optimized VMI energy level and routine CTE for CD and active CD. Statistical analysis for all data was conducted.

**Results:**

Objective and subjective imaging evaluations showed the best results at 60 keV for VMIs. The CT values of the normal group, active subgroup, and CD group during the small intestinal phase at routine 120 kVp or 60 keV VMI had significant differences. The diagnostic efficacy of an iodine map was the best when NIC = 4% or fat value = 45.8% for CD, whereas NIC < 0.35 or the fat value < 0.38 for active CD. The combined routine CTE and optimized VMI improved the diagnostic efficacy (*P* < 0.001).

**Conclusions:**

VMI at 60 keV provided the best imaging quality on DECTE. NIC and fat value provided important basis for active CD evaluation. Routine CTE combined with VMI at 60 keV improved the diagnostic efficiency for CD.

**Supplementary Information:**

The online version contains supplementary material available at 10.1186/s12880-021-00716-y.

## Background

Crohn’s disease (CD), one of the two main subtypes of inflammatory bowel disease (IBD), frequently and transmurally affects any segment of the digestive tract, particularly the walls of the terminal ileum and adjacent colon. The pathogenesis of CD remains unclear, and may be caused by multiple factors such as the environment, heredity, colonic microorganisms, the immune system, as well as other yet-unknown ones [1]. The prevalence of CD in China has increased in recent years owing to a change in diet [2]. The pathological characteristics of CD are identified by thickened walls and lumen stenosis of the intestine [3]. Moreover, because CD often presents itself through recurrence and remission over the lifetime of a patient, an assessment of the illness conditions is essential for diagnosis, treatment, and prognosis [4].

Standard measurements used in imaging examinations have been proposed to evaluate the activities of CD patients. Owing to the inconvenience and invasiveness of endoscopy, multi-slice computed tomography enterography (MSCTE), which is less expensive than magnetic resonance enterography, has become a popular and suitable way for a routine monitoring in CD patients [5–7]. Although the extent of the enhancements in the field of MSCTE has been closely connected with CD activities, there are many overlaps between the imaging characteristics of CD and other abdominal lesions [8–10]. In addition, intramural fat may cover some signs visible from an MSCTE, or the site of a hemorrhage may confuse the enhancement of MSCTE [11]. To avoid a misdiagnosis of CD, a distinguishable and clear image of the lesions is essential for a fast and accurate diagnosis.

Dual-energy CT (DECT) is an imaging technique based on data acquisition at two different energy settings, which can be used to diagnose lesions with higher quality and a more accurate quantity compared with a routine CT [12]. Dual-energy CT enterography (DECTE) is confirmed to have prominent ability to recognize the intestinal wall [13]. DECTE can not only obtain an imaging quality and diagnostic efficiency similar to that obtained in a routine CT [14, 15], but also significantly reduced both the radiation dose and number of CT scans [16, 17], offering unique advantages in the diagnosis and evaluation of activities in CD patients, especially young patients. DECTE can be used to not only quantitatively evaluate the degree of inflammation through the absolute iodine value (iodine map) of the enhancement based on an energy spectrum purification in an intestine with CD, but also improve the image contrast of normal blood supply vessels using virtual monochromatic imagings (VMIs) to clearly depict the boundary between an inflamed intestine and normal intestinal tissue [18]. DECTE can also clearly display the proliferative small vessels below grade 4 in low keV images and iodine maps, and they can be easily differentiated from the superior mesenteric artery (SMA) with an improved diagnostic efficiency [15]. Darras et al. showed the advantages and accuracy of using DECT in an intestinal obstruction study [19]. However, the diagnostic value of DECTE in CD has not been fully clarified.

In this retrospective study, we performed VMI reconstruction at several different energies and investigated the optimized kiloelectron volt (keV) for VMIs in DECTE. Then, we retrospectively explored the significance of the optimized keV of VMIs combined with iodine map in DECTE for an evaluation of the intestinal walls and vessels of patients with CD. Furthermore, the diagnostic efficacy of iodine maps for CD and active CD was analyzed, and that of DECTE + VMIs and a routine CTE was compared. A flowchart of our study is shown in Additional file 1: Figure S1. Our findings will provide a theoretical basis for improved diagnosis of CD.

## Methods

This study was approved by the Ethics Committee of Affiliated Hospital 2 of Nantong University (2019KW022), and the requirement to obtain informed written consent was waived. All methods were carried out in accordance with relevant guidelines and regulations.

### Patients

In a study on the optimized keV of VMI, the clinical and imaging data of 220 patients with DECTE in our hospital were collected from July 2018 to January 2019. The inclusion criteria of this study were as follows: (1) patients with a normal small intestine as verified through an endoscopy and complete clinical data; (2) patients with normal liver and kidney function, and without an allergy history of iodine or contraindications of contrast medium; (3) patients who underwent a DECTE test; and (4) patients with an intestinal filling score greater than 3. The intestinal filling score was defined as previous described [20, 21]. Finally, 72 patients (40 males and 32 females) ranging from 19 to 76 years old with an average age of 41.89 ± 17.28 met the inclusion criteria.

For an evaluation of the diagnostic efficacy of DECTE iodine map at the optimized VMI energy level and routine CTE for CD, we collected the clinical and imaging data of 92 patients staying in our hospital from June 2018 to December 2019, with a suspected disease in their small intestine. The inclusion criteria were the same as in the above study. However, the exclusion criteria of this study were as follows: (1) patients below 18 years of age; (2) patients with a pregnancy or serious underlying disease; (3) patients with an allergy history of iodine; and (4) patients with liver or kidney dysfunction. In addition, a total of 24 patients did not meet the inclusion or exclusion criteria. There were 12 patients without DECT data, 8 patients without complete clinical data, and 4 patients with a severe underlying disease. Finally, 68 patients were enrolled in the study, including 35 patients who were confirmed as having a normal small intestine through a close follow-up and 33 patients who were diagnosed as having CD based on endoscopy or previous pathology.

### DECTE

The patients were asked to avoid food for 4 or 8 h prior to the examination. Before preparing the intestinal filling, it was necessary to determine whether there were contraindications of the intestinal filling. One hour before the DECTE, those without contraindications, including intestinal obstruction or poor tolerance for isotonic mannitol were orally administrated with 2000 ml of 2.5% isotonic mannitol at an interval of 15 min (approximately 500 ml each time). Ten minutes before DECTE, those without contraindications for anisodamine (e.g. increased intracranial pressure and acute cerebral hemorrhage; glaucoma; prostatic hypertrophy; fresh fundus hemorrhage; malignant tumors; and pregnancy) were intramuscularly injected with 10 mg of per dose, prior to examination, to enhance the effect of the intestinal filling.

All CT examinations were performed through a dual-source DE unit (Somatom Force, Siemens Healthcare, Forchheim, Germany). A DECTE scan of each patient from the top of the diaphragm to the pubic symphysis was conducted. As the scanning parameters, a ball tube with tumor voltage of 90 kVp and tube current of 230 mAs, another ball tube with tube voltage of Sn150 kVp and tube current of 90 mAs, a fusion coefficient of 0.7, a pitch of 0.9, a slice thickness of 1 mm, and a slice spacing of 1 mm were applied [12]. First, a plain scan was conducted, and iopramine was injected into the anterior vein of the right elbow (at a concentration of 350 mg/ml). The scanning was auto-started at the arterial phase once the abdominal aorta arrived at the threshold (100 Hu), or at small intestine phase (approximately 40 s after injection), or at the venous phase (approximately 75 s after injection). All patients underwent CT scanning at all three phases.

### Image reconstruction

The SMA was evaluated through images of the arterial phase, and the original data were transferred to a Siemens Syngovia post-processing workstation. The images were reconstructed on the coronal plane of the SMA, with the maximum intensity projection (MIP) parallel to the trunk of the SMA (the thickness of the slice was 10.0 mm and the interval between slices was 5.0 mm), and a new single-energy software (Dual Energy Monoenergetic Plus, Mono + , Siemens Healthcare, Forchheim, Germany) was used to reconstruct the MIP images at 40–90 keV with increments of 10 keV and six groups of VMIs (Additional file 2: Figure S2). At the same time, a group of images at 120 kVp were obtained, which were the mixed images reconstructed with a linear fusion coefficient of 0.7 from the 90 kVp image scanned by a tube of dual-energy CT and the Sn150 kVp image scanned by another tube using Dual Energy Vascular.

The intestinal wall was evaluated using routine coronal images of the small intestine for a post-processing analysis of a multi-planar reformation (MPR) reconstruction. For the ileum at a distance of 1–5 cm of ileocecal (the thickness of the slice was 3.0 mm and the interval between slices was 3.0 mm), a set of routine images at 120 kVp, and six groups of VMIs were reconstructed using post-processing software (Additional file 3: Figure S3).

The diseased intestinal wall was evaluated using images taken at the intestinal phase, and the original dual energy data were transferred to Siemens Sygovia post-processing workstations. Routine coronal images were processed through an MPR reconstruction (the thickness of the slice was 3.0 mm, and the interval between slices was 3.0 mm) at 60 keV. In addition, the coronal plane of the SMA images was processed as an MPI reconstruction (the thickness of the slice was 10.0 mm, the interval between slices was 5.0 mm) at 60 keV. The two VMIs of the diseased intestinal wall and the SMA on the DECTE were adjusted to 100% using liver nailfold videocapillaroscopy (NVC) to obtain the complete iodine maps [12].

### Objective analysis of images

After the original images were magnified by three times, the region of interest (ROI) in the images was selected to avoid plaque and calcification affecting the measurements. The ROI was placed at the beginning of the SMA (area of 0.1 cm^2^) and gluteus maximus (area of 0.3 cm^2^). The CT values and noises with standard deviation (SD) were measured. For each measurement, the average values of three slices were obtained from the adjacent upper and lower slices to reduce the error. We then measured these magnified images again a week later. The calculated formulas of the signal-to-noise-ratio (SNR) and contrast-to-noise-ratio (CNR) were as follows: SNR (blood vessel) = vascular CT/SD value; CNR (blood vessel) = (vascular CT value—CT value of gluteus maximus muscle)/SD value of gluteus maximus muscle [22]; SNR (intestine) = intestinal CT/SD value; and CNR (intestine) = (intestinal CT value-CT value of gluteus maximus)/SD value of gluteus maximus muscle [22].

In the overall iodine maps, which were used as references for the endoscopy and clinical diagnosis, the ROI was selected at the thickest level of the diseased intestinal wall for measuring the absolute iodine value and fat value, and selected at the bifurcation of abdominal aorta and iliac vessels for measuring the iodine value as much as possible. The ROI was set as a circle covering approximately 60–90% of the diseased intestinal wall. When the enhancements of the images between slices are different, the ROI selection should avoid the region with different enhancements in the images. Moreover, it was better to set the ROI in the obvious lesion of the transverse and coronal directions at the mesenteric side and avoid the necrosis, calcification, and vessels. Compared with the control measurement of the normal intestinal wall, every index was measured at different directions and slices three times [23].

The calculated formula of the normalized iodine concentration (NIC) was as follows: NIC (mg/ml) = intestinal wall iodine concentration/iodine concentration near the iliac bifurcation of the abdominal aorta [23].

### Subjective image analysis

Images of SMA and its branches and intestinal wall were mainly observed. For the quantitative scores of blood vessel images, a five-component table [11] was used (Additional file 5: Table S1). To measure the grades of the blood vessels, we utilized an artificial intelligence (AI) model test through a constructed U-net model to segregate the SMA, and obtained images that clearly show the continuous blood vessels and the semi-automatically quantified grades of the SMA. Meanwhile, this study is mainly focused on images of vascular branches at Grade 4 and below (Additional file 4: Figure S4). According to the European CT Image Quality guidelines [24], the standard evaluation criteria for the subjective imaging quality and noise of the SMA and intestinal wall are shown in (Additional file 5: Table S2).

According to the latest expert consensus and references [25], the diagnostic criteria of the DECTE imaging evaluation for CD are as follows: intestinal wall thickening, intestinal wall enhancement and stratification, a mesenteric lymph node (MLN) enlargement, mesenteric exudation, straight small vessel hyperplasia (straight comb sign), at least three positive characteristic imaging signs of intestinal obstruction, peripheral abscess, fistula, and ascites. The quantitative parameters of DECTE images for evaluating the activity of CD are listed [26] in Additional file 5: Table S3. The intestinal images of a routine CTE at 120 kVp and DECTE + VMI at 60 keV using an iodine map were evaluated by two radiologists with 10 and 5 years of experience, respectively, who were blinded to the imaging parameters, clinical materials, and first pathological results. The two radiologists subjectively evaluated the imaging characteristics of the intestinal and extra-intestinal lesions and complications. Disagreements in the imaging evaluation between two radiologists were resolved by consensus.

The reconstructed images of the SMA and intestinal wall were double-blindly evaluated by two observers who were radiologists with 3 and 5 years of experience for imaging evaluation, respectively, who independently scored the images. The consistency between the two radiologists was evaluated based on an intraclass correlation coefficient (ICC) [12]. An ICC value of < 0.20 was considered to show poor confidence; 0.21–0.40, slight confidence; 0.41–0.60, moderate confidence; 0.61–0.80, high confidence; and 0.81–1.00, full confidence.

The quantitative parameters of the iodine map with statistical significance were drawn as the receiver-operating characteristic (ROC) curves, and the area under the curve (AUC) was used to evaluate the diagnostic efficacy of NIC of the diseased intestinal walls. The value of AUC ranged from 0.5 to 1.0, which was considered as a poorly approached truth at 0.5 and a perfectly approached truth at 1.0. However, the ROC curves were without any predictive significance when the AUC was 0.5. We further calculated the specificity, sensitivity, and optimal threshold for a differential diagnosis of the activity of the intestinal walls.

### Statistical analysis

All data were processed using SPSS 22.0 (IBM) statistical software. A Kolmogorv-Smirnov test was used to analyze the normal distribution of the data. The mean ± SD was used for data with a normal distribution, which was compared using a one-way ANOVA among groups, and was multiply compared using a Tukey test among every two groups. The data without a normal distribution (such as the CT value, SNR, and CNR) were expressed using the median ± quartile interval, and compared using a nonparametric Kruskal Wallis H test among groups, with a multiple pairwise comparison conducted among every two groups. The frequencies of the rating scales according to different physicians were compared using a paired χ2 test. A *P* value of < 0.05 was considered to be statistically significant.

## Results

### Screening of the optimized VMI energy level

#### Baseline of patients

A total of 72 patients (40 male and 32 female) with negative CTE were enrolled for investigation of the optimized keV of VMI in DECTE. The patients were 19 to 76 years old with a mean age of 41.89 ± 17.28. Clinical baselines of 72 patients with negative CTE is shown in Additional file 5: Table S4. 

#### Objective and subjective imaging evaluations showed the best results at 60 keV

The objective and subjective imaging evaluations of SMA are shown in Table 1. The objective imaging evaluation of SMA revealed that there were no significant differences in CNR and SNR between 60 keV group and 40 or 50 keV groups. Moreover, the CT value, CNR, and SNR of SMA at 60 keV were higher than that at the 70–90 keV groups. Subjective evaluation of SMA also showed that there was no significant difference in the number of branching vessels and imaging sharpness between 60 and 40 keV or 50 keV groups. Notably, the number of branching vessels and imaging sharpness in the 60 keV group were higher than those in both the 70–90 keV and 120 kVp groups (*P* < 0.001). Among these groups, the 60 keV group showed the best overall imaging quality with a significant difference (*P* < 0.05).

**Table 1:** Comparisons of subjective and objective evaluation parameters of SMA during arterial phase

Group	Objective evaluation parameters			Subjective evaluation parameters		
	CT value	CNR	SNR	Number of vascular branches ^*^	Overall image quality score ^*^	Image sharpness score ^*^
40 keV	1254.75 ± 235.47^a^	41.26 ± 14.62^a^	21.36 ± 7.14^b^	4.17 ± 0.81^a^	4.29 ± 0.32^b^	4.92 ± 0.28^a^
50 keV	831.33 ± 155.31^b^	37.18 ± 13.21^a^	21.20 ± 7.11^b^	4.14 ± 0.79^a^	4.34 ± 0..23^b^	4.91 ± 0..23^a^
60 keV	578.83 ± 105.95^c^	33.05 ± 12.22^a^	21.16 ± 7.29^b^	4.11 ± 0.78^a^	4.97 ± 0.20^a^	4.81 ± 0.23^a^
70 keV	421.19 ± 76.74^c^	28.5 ± 10.84^b^	20.92 ± 71.17^b^	3.38 ± 0.49^b^	4.12 ± 0.18^b^	4.14 ± 0.35^b^
80 keV	323.64 ± 63.03^d^	24.89 ± 9.78^bc^	19.03 ± 7.03^b^	3.33 ± 0.53^b^	4.03 ± 0.17^c^	3.98 ± 0.38^bc^
90 keV	249.22 ± 46.48^d^	18.45 ± 7.23^c^	14.92 ± 6.00^c^	2.36 ± 0.48^c^	4.06 ± 0.23^c^	3.03 ± 0.17^c^
120 kVp	431.81 ± 105.84^c^	21.74 ± 7.43^c^	26.55 ± 10.15^a^	3.36 ± 0.18^b^	4.08 ± 0.19^bc^	4.03 ± 0.29^b^
Z/F value	235.641	200.304	163.572	7.574	7.424	7.311
*P* value	< 0.001	< 0.001	< 0.001	0.817	0.828	0.716

"a, c, b, d" were multiple comparison marks. When marked with the same letter, it meant no significant difference between the two groups. When marked with different letters, it indicates the difference with statistically significant between the two groups. * subjective scores were expressed as mean ± standard deviation. CT: computed tomography; SMA: superior mesenteric artery; CNR: contrast-to-noise-ratio; SNR: signal-to-noise-ratio

The objective and subjective imaging evaluations of small intestinal wall are shown in Table 2. Similarly, the objective evaluation of small intestinal wall, the CT value, CNR, and SNR of the intestinal wall at 60 keV was significantly higher than that at 70–90 keV level (*P* < 0.05). For the subjective evaluation of small intestinal wall, the imaging noise, overall imaging quality, and sharpness scores at 60 keV were higher than those at 70–90 keV.

**Table 2:** Comparisons of subjective and objective evaluation parameters of small intestinal wall during small intestine stage

Group	Objective evaluation parameters			Subjective evaluation parameters		
	CT value	CNR	SNR	noise^*^	Overall imaging quality score *	Imaging sharpness score *
40 keV	149.44 ± 45.86^a^	3.18 ± 1.71^a^	5.08 ± 2.77^a^	4.76 ± 0.38^a^	4.57 ± 0.26^b^	4.91 ± 0.37^a^
50 keV	108.24 ± 31.03^b^	2.91 ± 1.45^a^	5.06 ± 2.54^a^	4.53 ± 0.71^a^	4.64 ± 0.23^b^	4.84 ± 0..53^a^
60 keV	83.24 ± 22.45^b^	2.74 ± 1.24^a^	4.93 ± 2.46^a^	4.38 ± 0.59^a^	4.91 ± 0.61^a^	4.27 ± 0.97^a^
70 keV	67.79 ± 17.47^c^	2.54 ± 1.14^b^	4.83 ± 2.14^b^	4.11 ± 0.78^b^	4.51 ± 0.37^b^	4.12 ± 0.68^b^
80 keV	57.59 ± 14.18^cd^	1.89 ± 1.05^c^	4.73 ± 2.17^b^	3.36 ± 0.85^c^	3.88 ± 0.28^c^	3.43 ± 0.27^c^
90 keV	51.15 ± 12.22^d^	1.52 ± 0.95^c^	4.52 ± 1.49^c^	3.17 ± 0.41^c^	3.73 ± 0.37^c^	3.06 ± 0.23^c^
120 kVp	61.18 ± 14.94^c^	2.74 ± 1.33^b^	4.55 ± 1.15^c^	3.96 ± 0.48^b^	4.13 ± 0.33^b^	4.28 ± 0.29^a^
Z/F value	185.041	98.104	82.172	5.164	5.221	5.111
*P* value	< 0.001	< 0.001	< 0.001	0.627	0.618	0.726

"a, c, b, d" were multiple comparison marks. When marked with the same letter, it meant no significant difference between the two groups. When marked with different letters, it indicates the difference with statistically significant between the two groups. * subjective scores were expressed as mean ± standard deviation. CT: computed tomography; CNR: contrast-to-noise-ratio; SNR: signal-to-noise-ratio

Collectively, objective and subjective imaging evaluations showed the best results at 60 keV.

#### Consistency of subjective imaging evaluations using two observers

The consistency analysis of the subjective evaluation parameters of the images between patients with and without CD indicated that the ICC of the imaging quality was consistent by two observers (ICC = 0.82) as well as the ICC of the branches of vessels and the imaging sharpness (ICC = 0.76 and 0.80, respectively). Significant differences were found in the rating scales of the small intestinal wall and the branching vessels (*P* < 0.001) with mid-level ICC values (ICC = 0.51 and 0.59, respectively) (Additional file 5: Table S5).

### Analysis the diagnostic efficiency of DECTE iodine map and routine CTE for CD

#### Baseline of patients

A total of 68 patients (45 males and 23 females) with a mean age of 38.27 ± 15.10 years were included for an evaluation of the diagnostic efficacy of DECTE iodine map at the optimized VMI energy level combined with routine CTE. There were 35 patients with an onset of abdominal pain verified without any intestinal lesions through a clinical close follow-up (24 males and 11 females). The age of these 35 patients ranged from 18 to 65 years and the median was 35. The other 33 patients were clinically or pathologically diagnosed as having CD (21 males and 12 females). The age of these 33 patients ranged from 16 to 55 years and the median was 27. Among the 33 patients with CD, 30 patients received the first clinical visit and endoscopic examination, and 10 patients received medical treatment before CTE examination. Finally, according to the clinical comprehensive evaluation, 18 of the 33 patients were in an active stage and 15 patients were in a remission stage. All lesions were involved in the intestinal wall of these patients (Table 3).

**Table 3:** Clinical baselines of 33 patients

Parameters	Contents
Ages	The age ranged from 16 to 55 years old with a median of 27 years old
Gender (female: male)	12: 21
Treatment	10 cases were treated by internal medicine and 23 cases were not treated
Involvement of the intestinal segment (cases)	Jejunum and ileum: 8
	Ileocecal: 25
	Colon: 15 All involved: 15
Degree of activity (cases)	Active stage: 18 Remission stage: 15
Mean of CRP	2.6 mg/dL (0.88 ~ 3.31 mg/dL)

#### Consistency of subjective imaging evaluations using two observers

**Fig. 1:**
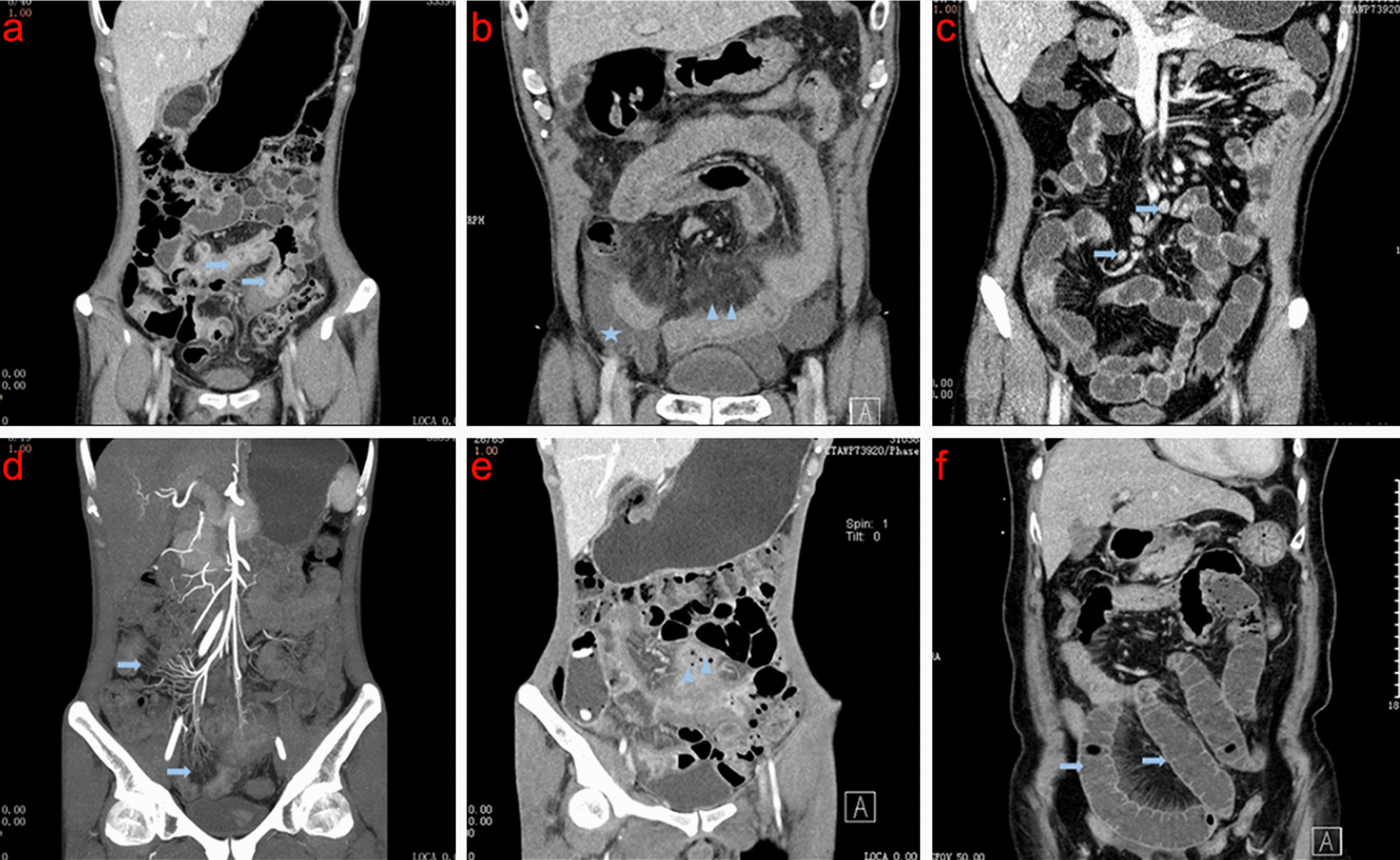
CTE signs and images of CD. **a** Intestinal wall thickening (arrow), **b** mesenteric exudation (arrow) and peritoneal effusion (pentagram), **c** MLN enlargement (arrow), **d** proliferation of straight small vessels and “ulnar comb sign” (arrow), **e** fistula forming abdominal abscess (arrow), and **f** secondary intestinal obstruction of CD (arrow)

The eight imaging characteristics of CD are shown in Fig. 1. The consistency evaluation of imaging characteristics of CD by the two observers showed that the range of ICC was 0.78–1.00, indicating the high consistency. In another consistency evaluation of the diagnosis of CD conducted by the two observers, different opinions for three cases under routine CTE at 120 kVp + VMI at 60 keV, and four cases under routine CTE at 120 kVp, were elicited. The ICC of the two observers using different detections were 0.91 (95% CI, 0.49–0.90) and 0.88 (95% CI, 0.70–0.95), respectively, suggesting that the final diagnosis given by both observers was consistent. Finally, we also conducted a consistency evaluation of CD activity under a routine CTE at 120 kVp + VMI at 60 keV and a routine CTE at 120 kVp, and found that the ICC was 0.82 (95% CI, 0.80–1.00) and 0.76 (95% CI, 0.37–0.79), respectively, with different opinions for three and four cases (Table 4).

**Table 4:** Consistency of imaging characteristics, diagnosis of CD and activity of CD between the two observers

	Regular CTE at 120 kVp + VMI at 60 keV				Regular CTE at 120 kVp			
	Doctor B	Doctor A		ICC value (95% CI)	Doctor B	Doctor A		ICC value (95% CI)
		Yes	No			Yes	No	
Intestinal wall thickening	Yes	33	0	1.000 (0.63–0.91)	Yes	33	0	1.000 (0.42–0.86)
	No	0	0		No	0	0	
Intestinal wall enhancement	Yes	28	3	0.900 (0.440.081)	Yes	30	0	0.783 (0.37–0.71)
	No	0	5		No	1	3	
Mesenteric lymph node enlargement	Yes	23	10	0.821 (0.70–0.98)	Yes	21	0	0.866 (0.61–0.88)
	No	0	10		No	1	12	
Mesenteric exudation	Yes	30	0	1.000 (0.80–1.0)	Yes	29	0	1.000 (0.56–0.80)
	No	0	3		No	0	4	
Straight small vessel proliferation	Yes	28	0	1.000 (0.50–0.91)	Yes	27	0	0.886 (0.62–0.99)
	No	0	4		No	1	6	
Combined intestinal obstruction	Yes	2	0	1.000 (0.35–1.0)	Yes	1	0	1.000 (0.49–0.72)
	No	0	31		No	0	32	
Fistula	Yes	1	0	1.000 (0.65–0.88)	Yes	1	0	1.000 (0.70–1.0)
	No	0	32		No	0	32	
Ascites	Yes	3	0	1.000 (0.80–1.0)	Yes	2	0	1.000 (0.38–0.88)
	No	0	30		No	0	31	
Diagnosis of CD	Yes	28	2	0.91 (0.49–0.90)	Yes	31	2	0.88 (0.70–0.95)
	No	1	37		No	2	33	
Activity of CD	Yes	16	2	0.82 (0.80–1)	Yes	15	2	0.76 (0.37–0.79)
	No	1	14		No	2	14	

CD: Crohn’s disease; CTE: computed tomography enterography; VMI: virtual monoenergetic imaging; ICC: intraclass correlation coefficient; 95% CI: 95% confidence interval

#### Comparisons of diseased intestinal wall images in CD during the small intestinal phase of CTE

**Fig. 2:**
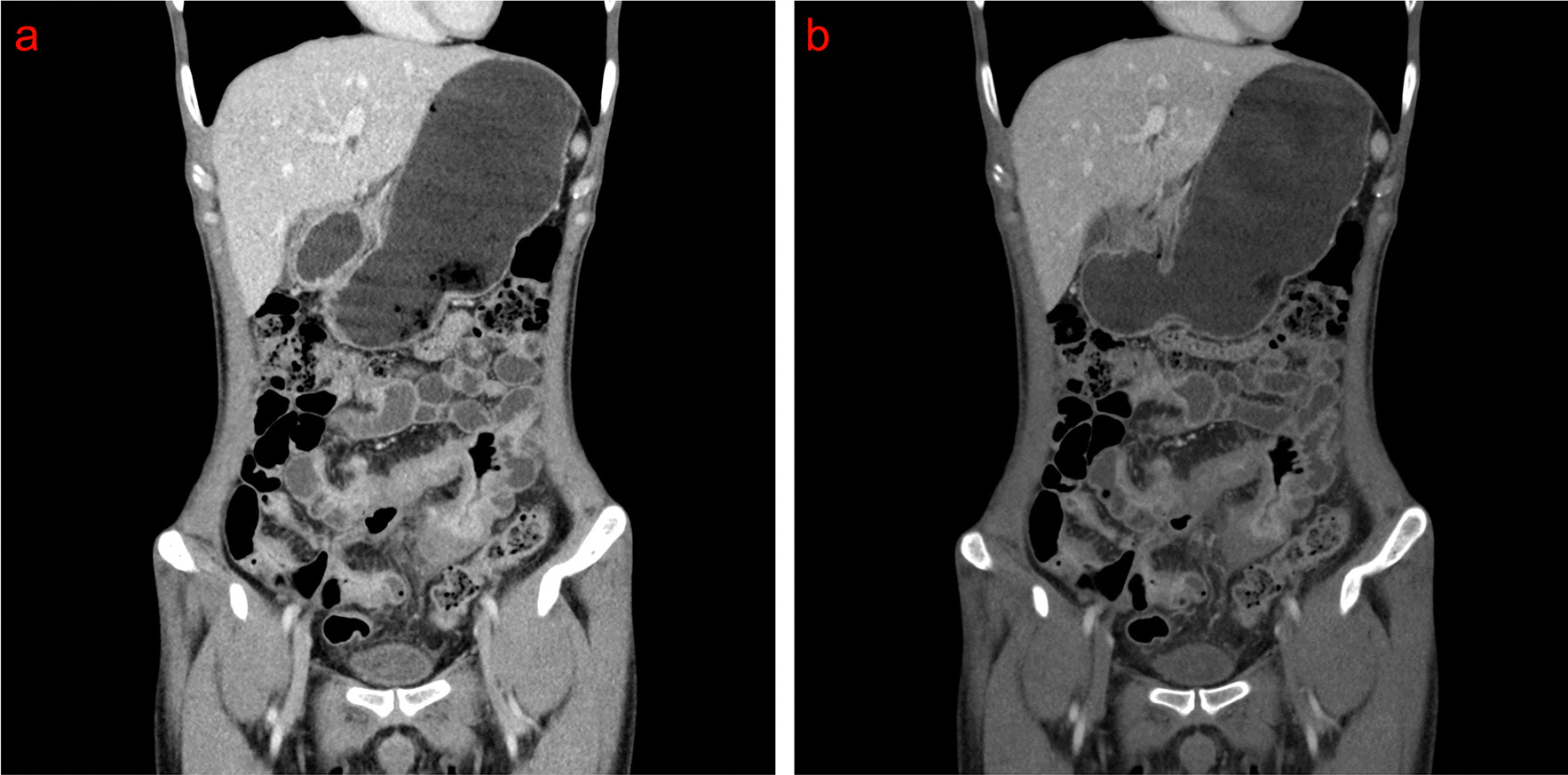
The intestinal wall images of a 36-year-old male CD patient at routine CTE at 120 kVp and routine CTE at 120 kVp + VMI at 60 keV. **a** Image at routine CTE at 120 kVp, **b** Image at routine CTE at 120 kVp + VMI at 60 keV

To visualize the clinical application of DECTE for CD diagnosis, the intestinal wall images of a CD patient at routine CTE at 120 kVp and routine CTE at 120 kVp + VMI at 60 keV are shown in Fig. 2. According to the diseased or normal small intestine, the patients were divided into CD and normal groups. The patients in the CD group were further divided into a remission subgroup and an active subgroup based on the CD activity. The differences in the quantitative characteristics of the intestinal segment, including the CT value (routine CTE at 120 kVp or VMI at 60 keV), NIC, iodine value, and fat value, were compared between the CD (remission and active groups) and normal groups.

For a quantitative characteristic of CTE, the CT values of the normal group, active subgroup, and CD group during the small intestinal phase at routine 120 kVp or 60 keV VMI had significant differences (*P* < 0.001). Among the parameters of the iodine map, the NIC and fat value in the CD group were higher than those of the normal group (*P* < 0.001), and the parameters of the iodine map in the active group were higher than the remission group (*P* < 0.001) (Table 5).

**Table 5:** Comparisons of parameters between different stage of diseased and normal intestinal wall images during the small intestinal phase of CTE

	Normal group	CD group (active subgroup)	CD group (remission subgroup)	Z/*F* value	*P* value
CT value (120 kVp)	51.28 ± 12.84	97.50 ± 40.29^a^	77.46 ± 14.51 ^b^	32.38	0.017
CT value (VMI at 60 keV)	83.57 ± 11.45	103.61 ± 18.12^a^	88.69 ± 22.43 ^b^	28.79	0.012
NIC	24.22 ± 10.16	54.62 ± 12.76 ^a^	36.23 ± 14.00 ^b^	342.58	< 0.001
Iodine density	3.21 ± 2.39	6.31 ± 1.85 ^a^	4.78 ± 1.48 ^b^	136.47	< 0.001
Fat fraction	35.38 ± 23.03	69.34 ± 17.24 ^a^	56.77 ± 13.20^b^	107.26	< 0.001

"a, b" were multiple comparison marks. When marked with different letters, it indicates the difference with statistically significant between the two groups. CD: Crohn’s disease; CT: computed tomography; CTE: computed tomography enterography; NIC: ormalized iodine concentration; VMI: virtual monoenergetic imaging

#### Diagnostic efficacy of DECTE iodine map

**Fig. 3:**
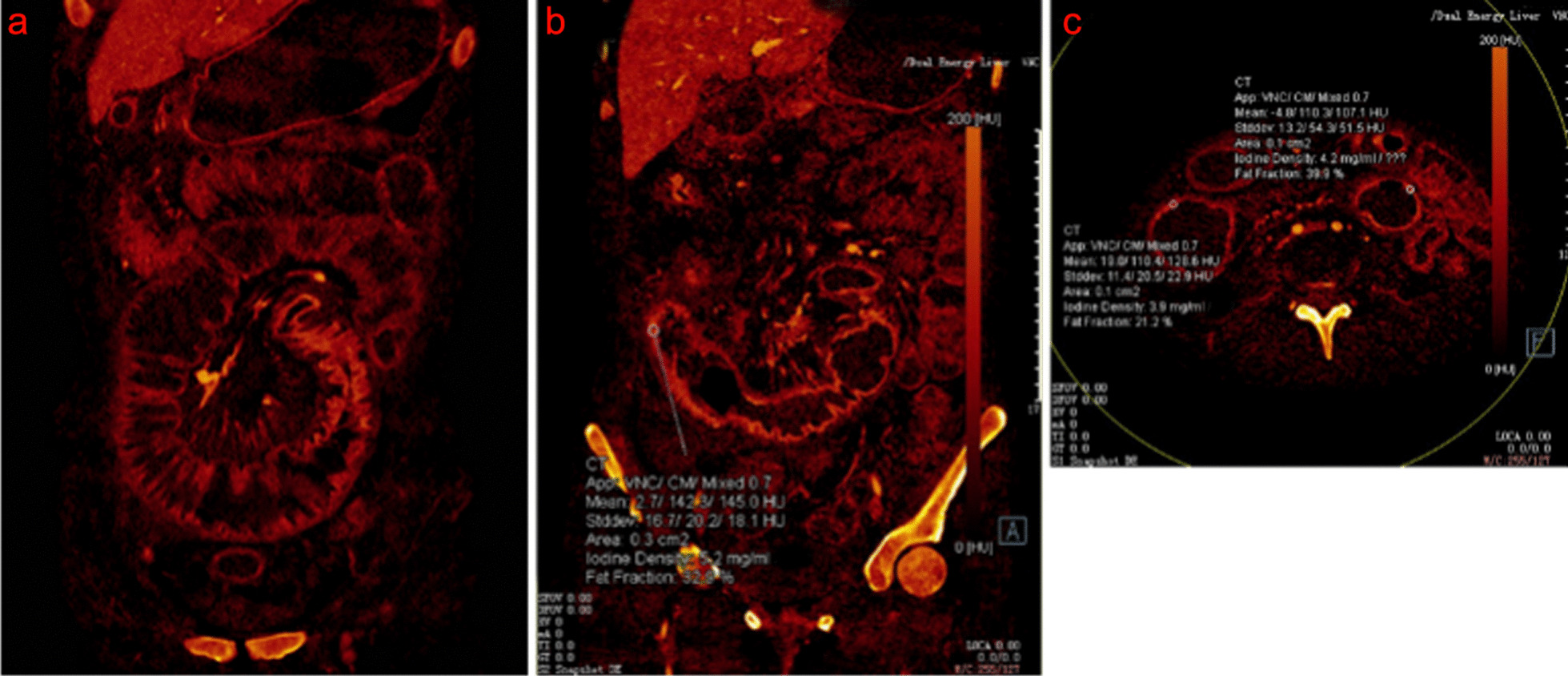
Parameter measurement of lesion on iodine map in intestinal wall of CD. A 38-year-old female was diagnosed with CD by endoscopy and clinical diagnosis (active stage). **a**, **b** the coronal position of the iodine map of the small intestine and **c** transverse position of the iodine map of the small intestine. **b** indicates that the ROI area was 0.3 cm^2^, the iodine value was 5.2 mg, the fat value was 32.9%, the ROI area was 0.1 cm^2^, the iodine value was 3.9 mg/ml, and the fat value was 21.2%. ROI: region of interest

**Fig. 4:**
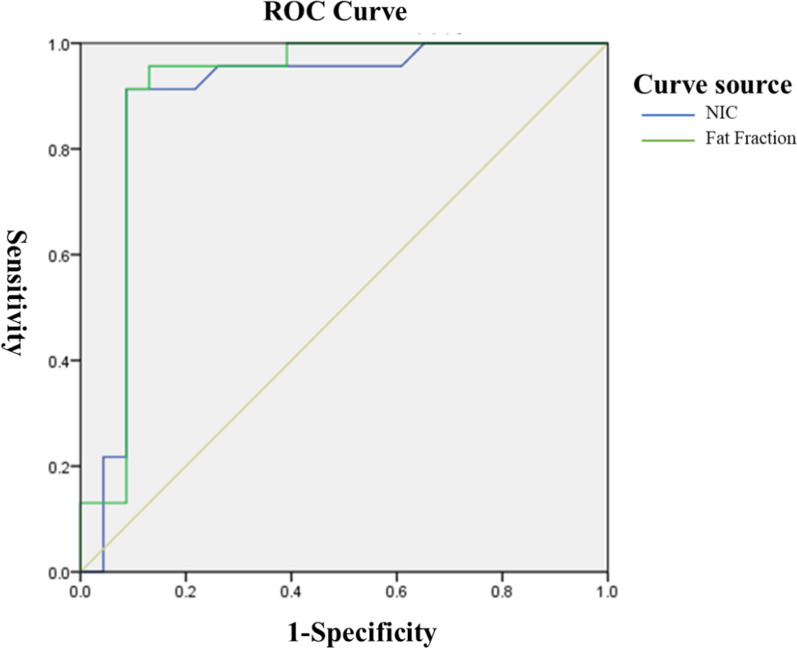
ROC curve of standardized iodine value and fat value in intestinal wall during small intestinal phase in diagnosed CD. In the ROC curve, when NIC < 0.83 (NIC = 4%) was used as the threshold and the maximum AUC was 0.89, the efficacy of differential diagnosis was the best. When a fat value of < 0.83 (fat value = 45.8%) was used as the threshold and the maximum AUC was 0.91, the efficacy of a differential diagnosis was the best

The ROC curve was used to analyze the diagnostic efficacy of NIC, and the fat value in the iodine map was evaluated by DECTE through a CD diagnosis (Fig. 3). There were 68 patients in this group, of which 33 were diagnosed with CD and 35 were normal. In the ROC curve, the maximum AUC was 0.89 when NIC < 0.83 (NIC = 4%) was used as the threshold. Thus, the best efficacy of the differential diagnosis was obtained with a specificity of 92.1% and a sensitivity of 90.9%. When a fat value < 0.83 (fat value = 45.8%) was used as the threshold, the maximum AUC was 0.91, and thus the best efficacy of a differential diagnosis was obtained with a specificity of 92.1% and a sensitivity of 90.9% (Additional file 5: Table S6 and Fig. 4).

Using the ROC curve to analyze the diagnostic efficacy of the iodine map evaluated using DECTE for active CD: There were 33 CD patients, among which 18 were in an active stage and 15 in a remission stage. In the ROC curve, according to the maximum point of the Youden index as the threshold, the maximum AUC is 0.72 when NIC < 0.35 was used as the threshold, and thus the efficacy of the differential diagnosis was the best with a specificity of 50.0% and a sensitivity of 85.2%. When a fat value < 0.38 was used as the threshold, the maximum AUC was 0.59, and thus the efficacy of a differential diagnosis was the best with a specificity of 59.1% and a sensitivity of 80.0% (Additional file 5: Table S7).

#### Diagnostic efficiency of VMI combined with routine CTE

**Fig. 5:**
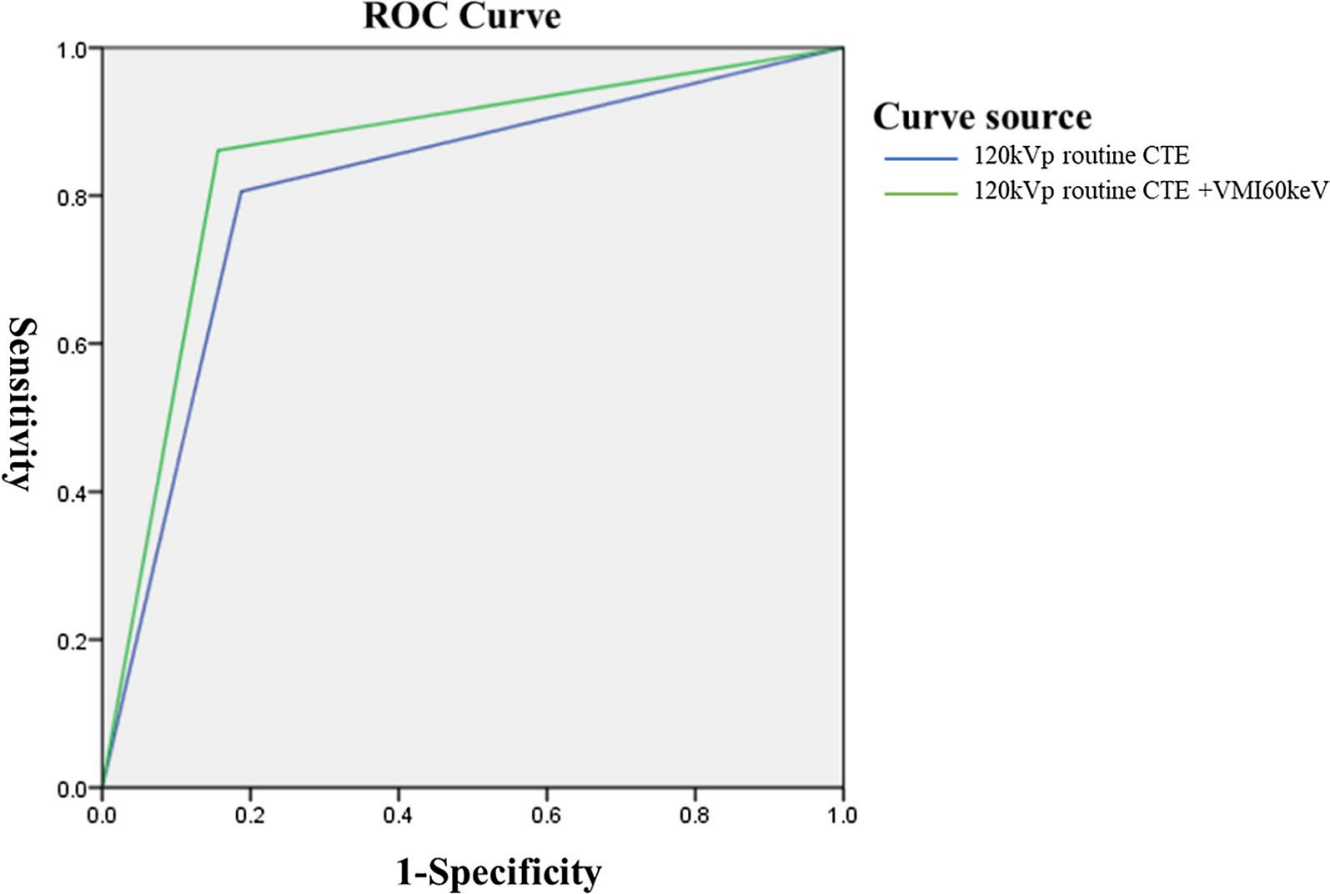
Comparison of diagnostic efficacy between routine CTE at 120 kVp + VMI at 60 keV and routine CTE at 120 kVp in CD diagnosis. A comparison of the ROC curve showed that the combined tests improved the diagnostic efficiency of CD with an increase in the AUC from 0.82 (routine CTE at 120 kVp) to 0.88 (routine CTE at 120 kVp + VMI at 60 keV) with statistical significance (*P* < 0.001)

Among the 68 patients, 33 cases were comprehensively diagnosed with CD through endoscope and clinical symptoms. Two radiologists using routine CTE at 120 kVp + VMI 60 keV diagnosed CD as a true positive in 29 cases, as a false positive in 4 cases, as a true negative in 33 cases, and as a false negative in 2 cases. In comparison, routine CTE at 120 kVp diagnosed CD as a true positive in 33 cases, false positive in 2 cases, true negative in 30 cases, and false negative in 5 cases. The sensitivity, specificity, positive predictive value, negative predictive value, and accuracy were 93.5%, 89.2%, 87.9%, 94.3% and 91.3%, respectively. The comparison of the ROC curve showed that the combined tests improved the efficiency of CD diagnosis with an increasing AUC from 0.82 (routine CTE) to 0.88 (combined tests) with statistical significance (*P* < 0.001) (Additional file 5: Table S8 and Fig. 5).

## Discussion

In the present study, we found that 60 keV was the optimized energy level to quantify images using DECTE, and the diagnostic efficacy of CTE at a routine 120 kVp linked with VMI at 60 keV was significantly improved in the diagnosis of CD, as proved by the increase in AUC from 0.82 (routine CTE) to 0.88 (combined tests).

Through an infusion of the contrast medium, enhanced CT can offer more detailed information on the thickness and bloodstream of a disease intestine when compared with the adjacent normal intestine [27]. The decline and elevated signal of enhancement can help with a differential diagnosis with inflammatory, infection, and metastasis scenes [28]. The intestine condition can be assessed using 120 kVp as a routine parameter, and CT attenuation is exhibited as white, gray, water halo, and black [29]. DECT is an innovative technique combined with enhanced CT and VMI, which aids in the reconstruction of a colorful or graded gray image at a lower energy level [30]. Moreover, an iodine map with a continuous enhancement of iodine is a practical tool to quantitatively measure the enhancement of the regions of interest in intestinal walls and to further provide evidence with strong confidence [29]. Iodine concentration, measured on detector-based DECTE, has been considered as a convenient and reproducible biomarker to detect disease activity in CD [11]. However, this process of quantification requires a skilled radiologist to select the appropriate threshold to determine the change in iodine concentration in a tissue, which is relevant with the diseased conditions of the intestine.

Anatomically, the branches of superior and inferior mesenteric artery supply the bloodstream of small intestine and form a circular branch of arcuate anastomosis in the mesentery. Wu et al. found that the degree of angiogenesis around the intestinal wall on the CTE was highly related to the activity of CD [31]. The small rectal vessels adjacent to the diseased intestinal wall proliferated and congested, showing the characteristic of a “comb-like” structure. Since endoscopy can only show the inter surface of intestinal walls, it is impossible to explore the proliferation of vessels around the intestinal wall or the extra-intestinal diseases. Meanwhile, CTE, which is applied for a three-dimensional observation and is post-processed to reconstruct the sagittal and coronal plane of the images, can solve the problems of endoscopy [32]. For the extra-intestinal complications, such as a blurred peri-intestinal fat space and mesenteric vascular hyperplasia, the sensitivity of CTE remains at 80%–100% with an obvious advantage in evaluating the condition and activity of CD [9].

Our results showed that CTE at 40–60 keV can better display the SMA without clear differences in imaging quality, which is consistent with the published literature [33]. Some scholars state that a new single-energy spectrum technology can reduce the noise of a low-keV single-spectrum image more significantly with a higher CNR [19]. In our study, there were no significant differences in the objective evaluation parameters (CNR and SNR) between 60 and 40 keV levels, and the overall imaging quality at 60 keV were higher than in the other energy level groups. Therefore, after a comprehensive consideration, we consider 60 keV to be the best energy level. Moreover, as 70 keV images quite compare to 120 kVp, simply reducing the tube voltage in SECT scans to 100 or 80 kVp may be an alternative to DECT.

Villanueva et al. found that the CTE measurement of the terminal ileum was closely related to the endoscopic results [34]. The enhancement of the intestinal wall on the CTE can predict the activity of CD inflammation with a high sensitivity of 90%, which reaches 80% for a visual assessment [35]. As is well-known, active inflammation is linked with more blood perfusion and accompanying iodine concentration, and thus the iodine map on DECTE can clearly indicate the activity of CD [36]. However, the efficacy of the fat value in the assessment of CD activity is low in our study, which may be explained as the proliferation of fat caused by a long-term repeated injury of the intestinal wall under CD conditions. Therefore, both NIC and the fat value should be practically considered in the diagnosis of CD.

In this study, we innovatively compared patients with and without CD through DECTE and conducted a comprehensive clinical diagnosis to protect the subjects from high doses of radiation. We then utilized a VMI in CTE to subjectively evaluate the images at the best energy level, which was appropriate for displaying the intestinal walls and vessels simultaneously. In addition, we utilized the U-net model to semi-quantitatively determine the branches of the SMA, which transferred the subjective evaluation to the objective index. Finally, we demonstrated the value of DECTE in a CD diagnosis through the reference threshold of the iodine maps, and the value of the MIP reconstruction of the SMA (the thickness of the slice was 10.0 mm and interval between slices was 5.0 mm) in the diagnosis of inflammatory vessel proliferation. However, there were some limitations to this study: (1) Evaluations in normal patients with multiple displays of normal intestine showed some deviation. (2) Subjective scores showed some bias for an evaluation of the intestinal wall. (3) The sample size of the CD patients was small, and subjective imaging evaluations were conducted by only two observers. Further studies with more participants and observers are needed to confirm our findings. (4) The concentration of iodine was influenced by the injection dose and patient status, requiring further evidence to verify these quantified data.

## Conclusion

In conclusion, CTE at 120 kVp linked with a VMI at 60 keV can better display the intestinal wall and blood vessels simultaneously. Meanwhile, the DECTE and quantitative NIC parameters are valuable for the diagnosis and evaluation of activity in CD patients, respectively. Finally, the combined use of VMI at 60 keV and conventional CTE at 120 kVp can improve the efficiency of CD diagnosis.

## Supplementary Information

**Additional file 1: Figure S1**. A flowchart of our study.

**Additional file 2: Figure S2**. Comparison of SMA images at different keV energy levels of VMI. (a)–(f) MIP images of SMA reconstructed at 40, 50, 60, 70, 80, and 90 keV, respectively. The thickness of the slice was 10.0 mm and the interval between slices was 5.0 mm.

**Additional file 3: Figure S3**. Comparison of normal intestinal wall images at different keV energy levels of VMI. (a)–(f) MPR images of intestinal wall reconstructed at 40, 50, 60, 70, 80, and 90 keV, respectively. The thickness of the slice was 3.0 mm and the interval between slices was 3.0 mm.

**Additional file 4: Figure S4**. Semi-automatically quantitative images of SMA at different energy levels of VMI.

**Additional file 5: Table S1**. Subjective quantitative scores of images of blood vessels. **Table S2**. Subjective evaluation of imaging quality and noise as 5-point score. **Table S3**. Quantitative parameters of DECTE images for evaluating the activity of CD. **Table S4**. Clinical baselines of 72 patients with negative CTE. **Table S5**. Consistency of the subjective evaluation parameters between the two observers. **Table S6**. Comparisons of efficacy in CD diagnosis between NIC and fat value of intestinal wall during the small intestine phase of CTE. **Table S7**. Comparisons of efficacy in active CD between NIC and fat value of intestinal wall during the small intestine phase of CTE. **Table S8**. Comparison of diagnostic value between routine CTE+ VMI60keV and routine CTE at 120 kVp in CD diagnosis.

## Data Availability

The data used to support the findings of this study are available from the corresponding author upon request.
