# Supplementary figures and images for: Efficiency of dual-energy computed tomography enterography in the diagnosis of Crohn’s disease
Source: BMC Med Imaging. 2021 Dec 3;21:185. doi: 10.1186/s12880-021-00716-y (PMC8642845; doi:10.1186/s12880-021-00716-y)

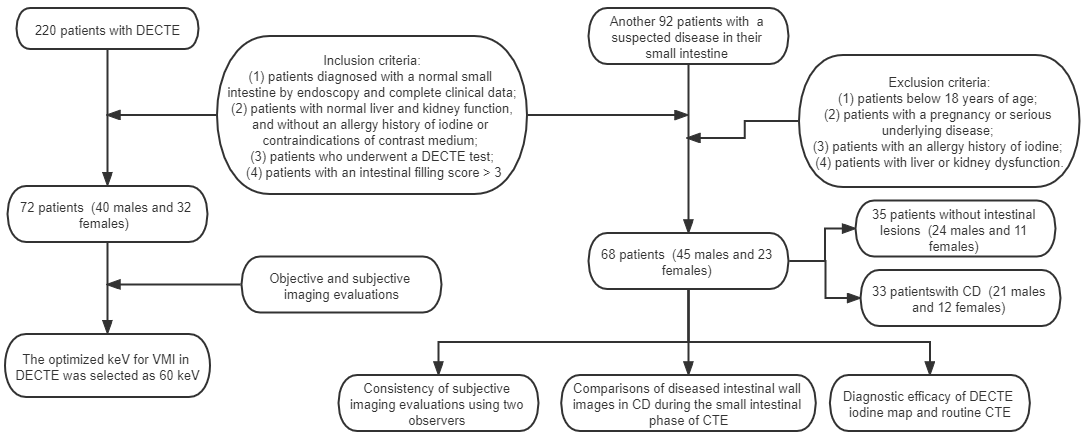

Supplement: Supplementary file 1 — Additional file 1: Figure S1. A flowchart of our study. [file 12880_2021_716_MOESM1_ESM.tif]

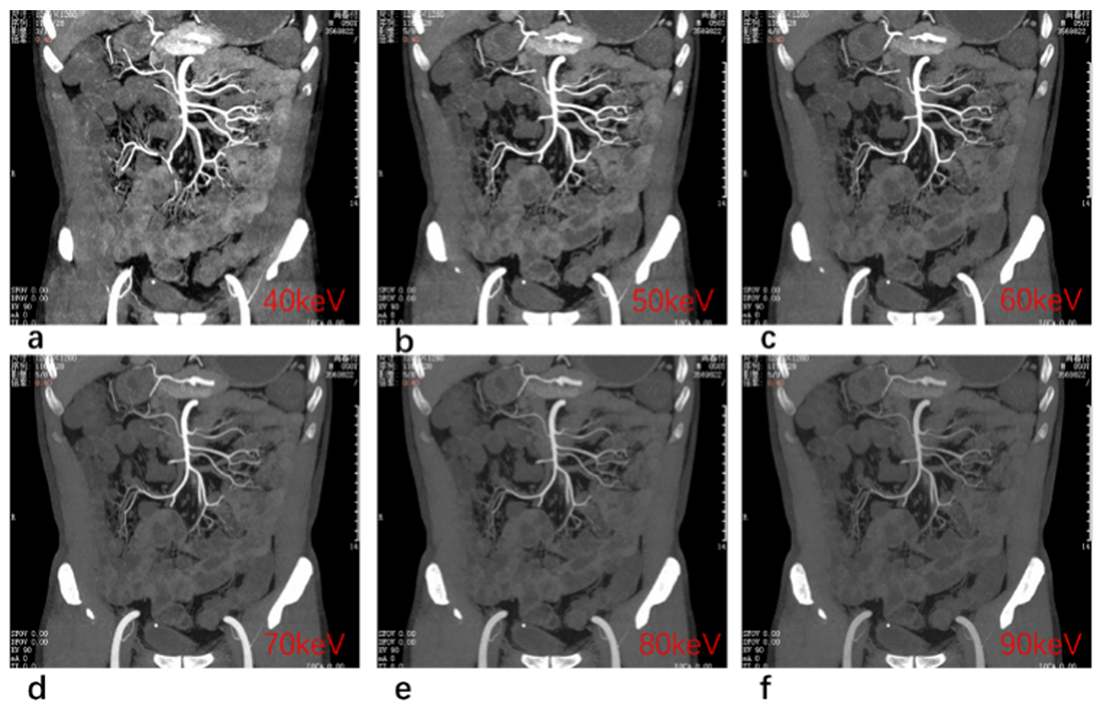

Supplement: Supplementary file 2 — Additional file 2: Figure S2. Comparison of SMA images at different keV energy levels of VMI. (a)–(f) MIP images of SMA reconstructed at 40, 50, 60, 70, 80, and 90 keV, respectively. The thickness of the slice was 10.0 mm and the interval between slices was 5.0 mm. [file 12880_2021_716_MOESM2_ESM.tif]

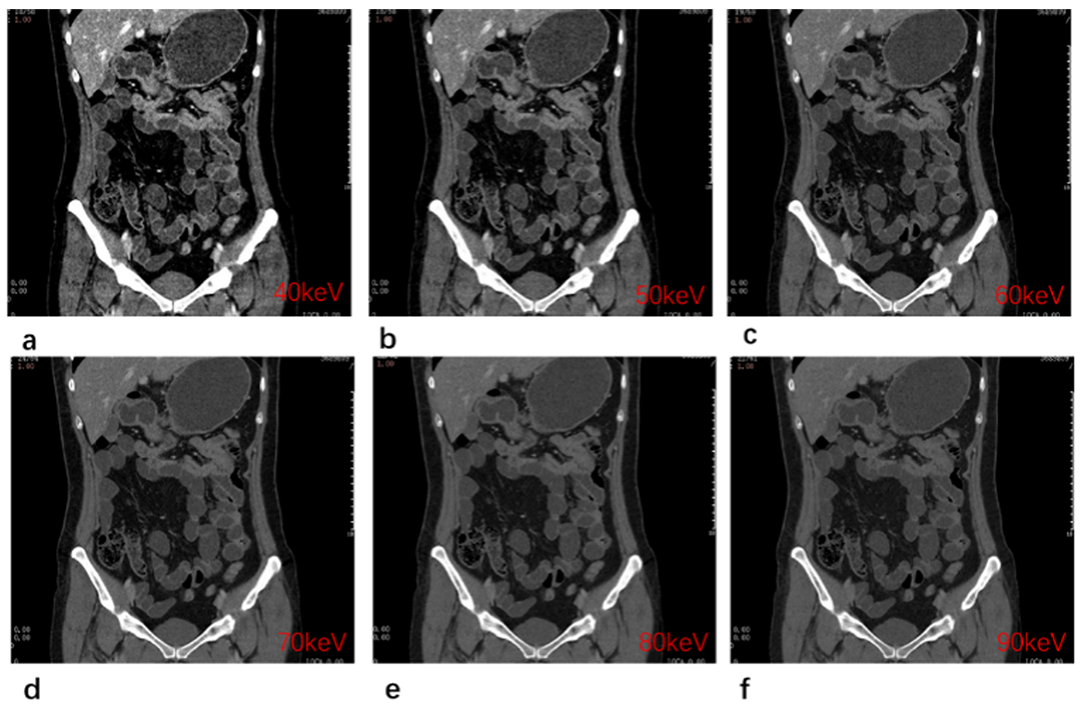

Supplement: Supplementary file 3 — Additional file 3: Figure S3. Comparison of normal intestinal wall images at different keV energy levels of VMI. (a)–(f) MPR images of intestinal wall reconstructed at 40, 50, 60, 70, 80, and 90 keV, respectively. The thickness of the slice was 3.0 mm and the interval between slices was 3.0 mm. [file 12880_2021_716_MOESM3_ESM.tif]

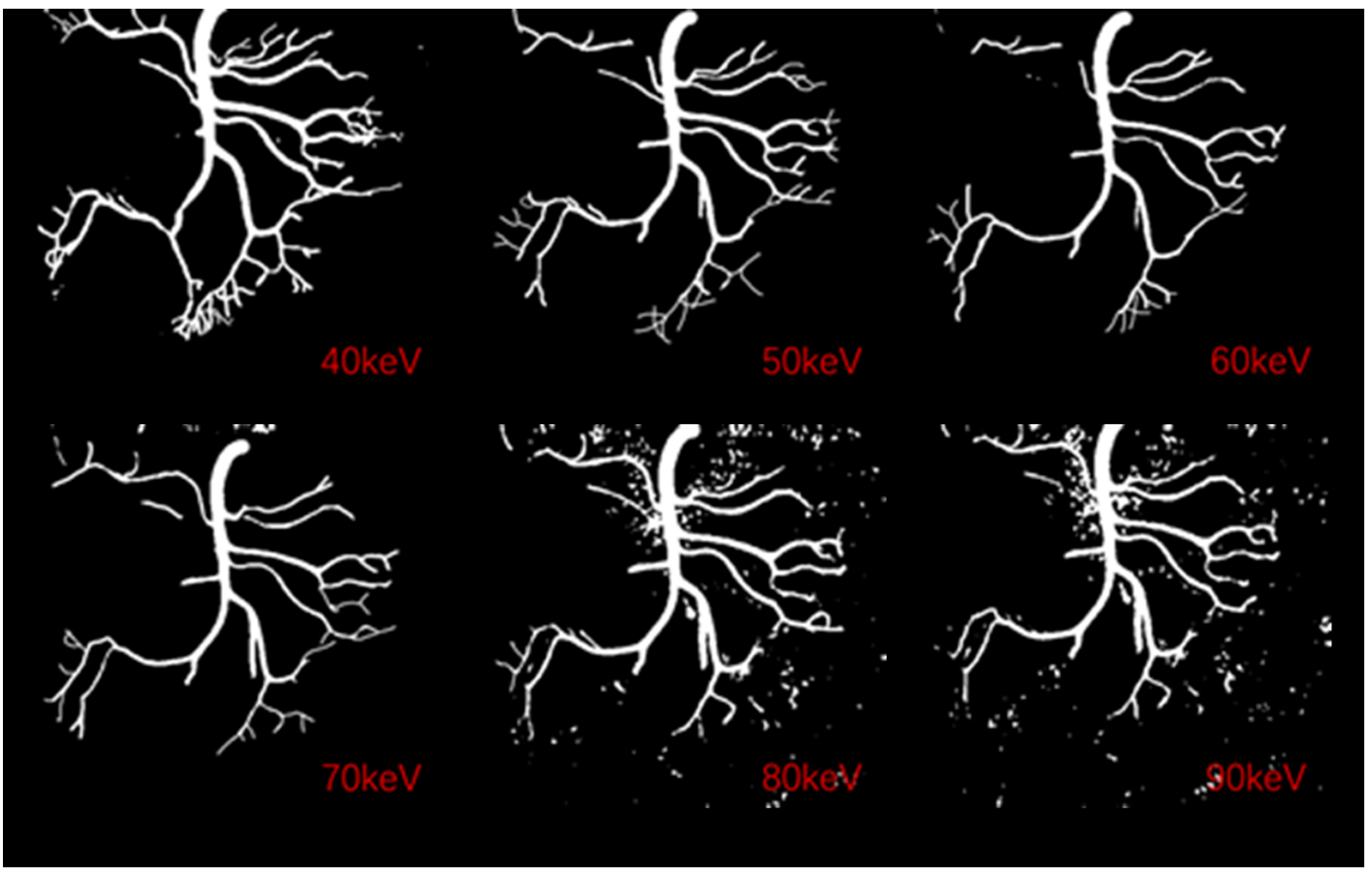

Supplement: Supplementary file 4 — Additional file 4: Figure S4. Semi-automatically quantitative images of SMA at different energy levels of VMI. [file 12880_2021_716_MOESM4_ESM.tif]
