# Supplementary material for: Efficiency of dual-energy computed tomography enterography in the diagnosis of Crohn’s disease
Source: BMC Med Imaging. 2021 Dec 3;21:185. doi: 10.1186/s12880-021-00716-y (PMC8642845; doi:10.1186/s12880-021-00716-y)
Supplement: Supplementary file 5 — Additional file 5: Table S1. Subjective quantitative scores of images of blood vessels. Table S2. Subjective evaluation of imaging quality and noise as 5-point score. Table S3. Quantitative parameters of DECTE images for evaluating the activity of CD. Table S4. Clinical baselines of 72 patients with negative CTE. Table S5. Consistency of the subjective evaluation parameters between the two observers. Table S6. Comparisons of efficacy in CD diagnosis between NIC and fat value of intestinal wall during the small intestine phase of CTE. Table S7. Comparisons of efficacy in active CD between NIC and fat value of intestinal wall during the small intestine phase of CTE. Table S8. Comparison of diagnostic value between routine CTE+ VMI60keV and routine CTE at 120 kVp in CD diagnosis. [file 12880_2021_716_MOESM5_ESM.docx]

**Table S1. Subjective quantitative scores of images of blood vessels**

| Scoring | Specific evaluation criteria |
| --- | --- |
| 1 point | Only Grade 1 branches were displayed, the shape was blurred and cannot be diagnosed. |
| 2 point | Grade 2 branches could be displayed with unclear shape and poor detail display. |
| 3 point | Grade 3 branches could be displayed, most of the blood vessels could be clearly displayed, a few of blood vessels could not be clearly displayed or evaluated |
| 4 point | It could display grade 4 branches, the shapes and details were clearly displayed. It could be evaluated, but was not ideal. |
| 5 point | The shape and details of blood vessels were clearly displayed, which could be evaluated accurately. |

**Table S2. Subjective evaluation of imaging quality and noise as 5-point score**

| Score | Overall imaging quality | Imaging sharpness | Noises |
| --- | --- | --- | --- |
| 1 point | Unable to diagnosis | Very blurry | Extensive noises |
| 2 point | Considerable impact to diagnosis | High blurry | Acceptable noises |
| 3 point | Moderate impact to diagnosis | Moderate blurry | Moderate noises |
| 4 point | Few impact to diagnosis | Little blurry | Few noises |
| 5 point | Diagnosis with high confidence | No blurry | No noise |

**Table S3. Quantitative parameters of DECTE images for evaluating the activity of CD**

| Parameters | Scores |
| --- | --- |
| Intestinal wall： |  |
| Intestinal length | 1 point: ≤5 cm, 2 point: >5 cm, 3 points: multi-segment |
| Intestinal thickness | 1 point: 3~4cm, 2 point: 4~5cm; 3 point: >5cm |
| Enhancement | 0 point: no enhancement, 1 point: single intestinal segment enhancement, 2 point: multiple intestinal segments |
| Extra-intestinal lesions and complications (3 points) | MLN enlargement, fat band sign (fat exudation around the intestinal wall), increase and thickening of small rectal vessels (comb sign), intestinal obstruction, peripheral abscess, fistula and ascites |
| Diagnostic criteria | ≥6 point: obviously active period, ≥12 point: severely active period |

DECTE: dual-energy computed tomography enterography; CD: Crohn’s disease

**Table S4. Clinical baselines of 72 patients with negative CTE**

|  | | Males (n = 40) | Females (n = 32) |
| --- | --- | --- | --- |
| Ages (years) | | 39.5 ± 12.3 | 42.9 ± 11.5 |
| Normal whole abdomen (cases) | | 6 | 4 |
| Adrenal adenoma (cases) | | 1 | 0 |
| Hepatic hemangioma (cases) | | 4 | 2 |
| Hepatic cyst (cases) | | 6 | 5 |
| Hepatic cyst with renal cyst (cases) | | 8 | 5 |
| Renal cyst (cases) | | 9 | 7 |
| Kidney stones (cases) | | 2 | 3 |
| Cholecystitis complicated with gallbladder stones (cases) | | 0 | 1 |
| Gallbladder stones (cases) | | 1 | 2 |
| Mesenteric panniculitis (cases) | | 0 | 1 |
| SMA origin variant (cases) | | 2 | 1 |
| SMA aneurysm (cases) | | 1 | 0 |
| Uterine fibroid (cases) | 0 | | 1 |

CTE: computed tomography enterography; SMA: superior mesenteric artery.

**Table S5. Consistency of the subjective evaluation parameters between the two observers**

|  | Doctor B  (frequency of scores) | Doctor A  (frequency of scores) | | | P value |
| --- | --- | --- | --- | --- | --- |
|  |  | 3 | 4 | 5 |  |
| SMA | 3 | 1 | 8 | 0 | <0.001 |
|  | 4 | 5 | 325 | 156 |  |
|  | 5 | 0 | 0 | 9 |  |
| Small intestinal wall | 3 | 0 | 0 | 0 | <0.001 |
|  | 4 | 0 | 126 | 46 |  |
|  | 5 | 0 | 68 | 262 |  |

SMA: superior mesenteric artery

**Table S6. Comparisons of efficacy in CD diagnosis between NIC and fat value of intestinal wall during the small intestine phase of CTE**

|  | AUC | Sensitivity | Specificity | Optimal value | *P* |
| --- | --- | --- | --- | --- | --- |
| NIC | 0.89 | 0.91 | 0.92 | 0.83 | <0.001 |
| Fat fraction | 0.91 | 0.91 | 0.92 | 0.83 | <0.001 |

CD: Crohn’s disease; CTE: computed tomography enterography; NIC: ormalized iodine concentration; AUC: area under the curve.

**Table S7. Comparisons of efficacy in active CD between NIC and fat value of intestinal wall during the small intestine phase of CTE**

|  | AUC | Sensitivity | Specificity | Optimal value | *P* |
| --- | --- | --- | --- | --- | --- |
| NIC | 0.72 | 0.85 | 0.5 | 0.35 | 0.034 |
| Fat fraction | 0.59 | 0.80 | 0.59 | 0.38 | 0.381 |

CD: Crohn’s disease; CTE: computed tomography enterography; NIC: ormalized iodine concentration; AUC: area under the curve.

**Table S8. Comparison of diagnostic value between routine CTE at 120 kVp + VMI at 60keV and routine CTE at 120 kVp in CD diagnosis**

|  | Routine CTE at 120 kVp +VMI at 60keV | Routine CTE at 120 kVp |
| --- | --- | --- |
| CD case (n=68) |  |  |
| Sensitivity | 93.5% (29/31) | 84.8% (28/33) |
| Specificity | 89.2% (33/37) | 85.7% (30/35) |
| Positive predictive value | 87.9% (29/33) | 84.8% (28/33) |
| Negative predictive value | 94.3% (33/35) | 85.7% (30/35) |
| Accuracy rate | 91.2% (62/68) | 85.3% (58/68) |

CD: Crohn’s disease; CTE: computed tomography enterography; VMI: virtual monoenergetic imaging.
